# MARMOSET MONKEYS AS A MODEL OF AGING

**DOI:** 10.1093/geroni/igz038.028

**Published:** 2019-11-08

**Authors:** Suzette Tardif, Corinna Ross

**Affiliations:** Southwest National Primate Research Center, San Antonio, Texas, United States

## Abstract

Interest in the New World Monkey, the common marmoset, as a nonhuman primate aging model is growing. Because marmosets have a fast maturation and short life span compared with more commonly used Old World monkey models, the aging research community began to explore the potential of this model species. In addition, the relative ease with which marmosets can be bred in a barrier environment enhances their value as a life-span model. Since that time, efforts to better define what aging actually looks like in marmosets has intensified. Important findings of the past decade include: (1) a refined definition of lifespan in this species and what affects age-specific survival; (2) assessments of age-related pathological changes; (3) development of functional phenotyping relevant to aging, such as activiyy, strength, body composition, cytokine profiling; (4) support of studies using the marmoset as a preclinical model to test intervention that may modulate the aging process.

